# Synergistic nanocoating with layer-by-layer functionalized PCL membranes enhanced by manuka honey and essential oils for advanced wound healing

**DOI:** 10.1038/s41598-024-71466-4

**Published:** 2024-09-05

**Authors:** Camilla Gallo, Joel Girón-Hernández, Daisy A. Honey, Edward M. Fox, Maria A. Cassa, Chiara Tonda-Turo, Irene Camagnola, Piergiorgio Gentile

**Affiliations:** 1 School of Engineering, Newcastle University, Newcastle Upon Tyne, NE1 7RU UK; 2 Department of Applied Sciences, Faculty of Health and Life Sciences, Northumbria University, Newcastle Upon Tyne, NE1 8ST UK; 3 Department of Mechanical and Aerospace Engineering, Politecnico Di Torino, 10129 Turin, Italy; 4 Polito BIOMed Lab, Politecnico Di Torino, 10129 Turin, Italy

**Keywords:** Layer-by-layer assembly, Electrospun membranes, Manuka honey, Essential oils, Wound healing, Biomedical engineering, Biomaterials

## Abstract

Chronic wounds represent a significant global health concern, statistically impacting 1–2% of the population in developed countries throughout their lifetimes. These wounds cause considerable discomfort for patients and necessitate substantial expenditures of time and resources for treatment. Among the emerging therapeutic approaches, medicated dressings incorporating bioactive molecules, including natural compounds, are particularly promising. Hence, the objective of this study was to develop novel antimicrobial dressings for wound treatment. Specifically, polycaprolactone membranes were manufactured using the electrospinning technique and subsequently coated with natural polyelectrolytes (chitosan as a polycation and a mixture of manuka honey with essential oils nanoemulsions as a polyanion) employing the Layer-by-Layer assembly technique. Physico-chemical and morphological characterization was conducted through QCM-D, FTIR-ATR, XPS, and SEM analyses. The results from SEM and QCM-D demonstrated successful layer deposition and coating formation. Furthermore, FTIR-ATR and XPS analyses distinguished among different coating compositions. The coated membranes were tested in the presence of fibroblast cells, demonstrating biocompatibility and expression of genes coding for VEGF, COL1, and TGF-β1, which are associated with the healing process (assessed through RT-qPCR analysis). Finally, the membranes exhibited excellent antibacterial activity against both *Staphylococcus aureus* and *Pseudomonas aeruginosa*, with higher bacterial strain inhibition observed when cinnamon essential oil nanoemulsion was incorporated. Taken together, these results demonstrate the potential application of nanocoated membranes for biomedical applications, such as wound healing.

## Introduction

Wounds can be defined as the disruptions of tissue continuity^1^, that undergo a healing process involving phases such as haemostasis, inflammation, proliferation, and remodelling^2^. Chronic wounds deviate from this process, leading to issues such as pathological inflammation^3^, impaired angiogenesis^4^, and the formation of drug-resistant biofilms^5^. These non-healing wounds result in significant patient discomfort and severe health problems, including disabilities and amputations^6^.

In 2022, the chronic wound care market size reached USD 16.7 billion, and it is estimated to grow to USD 25.1 billion by 2033^7^. Antimicrobial resistance (AMR) exacerbates the challenge, as pathogens develop resistance to antimicrobial agents, contributing to treatment failure and increasing infection severity. AMR has caused at least 1.3 million deaths worldwide, with associated costs of USD 4.6 billion annually^8^. Novel treatment solutions are urgently needed to address this pressing global healthcare issue^9^. Wound treatments typically include systemic therapies, local therapies, skin grafting, skin substitutes, and wound dressings (WDs)^4^. Wound dressings, designed to cover wounds and promote healing, prevent infections, manage exudates, ensure hydration and gas exchange, reduce pain, and improve aesthetics^10,11^. Various dressings, tailored to specific wound characteristics such as depth, dimension, location, and exudate amount, are available^12^. Traditional dressings, such as gauze and bandages, offer mechanical protection but may cause pain upon removal and require frequent changes. In contrast, advanced dressings, often produced using tissue engineering techniques, enhance biocompatibility and bioactivity, leading to improved healing outcomes^13^. Moreover, medicated WDs incorporate bioactive components or controlled-release systems, with recent advancements including automated WDs with decisional algorithms and drug-delivery actuators^14^.

Within the manufacturing techniques, electrospinning stands as a conventional method for producing membranes for tissue engineering. This method facilitates the creation of non-woven, randomly aligned substrates composed of interwoven ultrathin fibers, with diameters ranging from tens of nanometres to a few micrometres^15^. Additionally, the electrospinning allows adjustment of the membrane's porosity, shape, and composition to suit specific applications. It is recognized for its versatility, cost-effectiveness, and ease of implementation^16^. These membranes serve as ideal solutions for wound dressings because they offer mechanical support, fill cavities, act as barriers against contaminants, facilitate gas and liquid exchange, and can be biocompatible and biodegradable based on the chosen material. Moreover, their structure mirrors that of the extracellular matrix (ECM)^17^. Their high porosity and proper pore distribution enable efficient exudate absorption and biomolecule incorporation, with functionalization enhancing wound healing and antibacterial properties^18^.

A prominent surface functionalization method involves electrostatically driven Layer-by-Layer (LbL) assembly. This technique effectively integrates therapeutic agents, precisely controls the loading dose, and ensures favorable kinetics for local sustained release from the nanofibrous matrix. Simultaneously, it incorporates biomimetic elements that guide tissue regeneration^19^ The LbL method relies on the interaction between oppositely charged layers of polyelectrolytes^20^. It enables the creation of nanoscale coatings on various substrates with tailored properties, based on the integration of selected bioactive compounds, such as polymers, proteins, lipids, nucleic acids, nanoparticles, and metal oxides^21,22^. Notably, this does not compromise the film’s mechanical properties, or its final porosity. Researchers have explored combining electrospinning and LbL techniques for WDs applications, often replacing traditional antibiotics with natural antimicrobial agents. These electrospun membranes typically use biocompatible polymers such as biodegradable poly lactic acid (PLA), polycaprolactone (PCL), or PCL-based blends. They are, then, further enhanced through LbL deposition of natural antimicrobials, such as chitosan, antimicrobial peptides and tannic acid, along with typical wound healing promoters e.g., collagen, hyaluronic acid and heparin^23–25^. Recent studies have also delved into the LbL functionalization of silk fibroin nanofibrous mats, aiming to bolster their antibacterial and biocompatible attributes^26,27^.

The study addressed how to effectively functionalize biodegradable PCL electrospun membranes with a non-woven ECM-like structure at the nanoscale for wound dressing applications. Specifically, it investigated methods for surface functionalization through Layer-by-Layer assembly to create a multilayered nanocoating using natural antibacterial agents such as chitosan (CH) and Manuka honey (MH) as polyelectrolytes. The study also explored the impact of integrating nanoemulsions of tea tree essential oil (TEO) and cinnamon essential oil (CEO) into the polyanionic Manuka honey solution on enhancing the antibacterial activity of the wound dressing. The parameters employed for the preparation of the nanoemulsions were optimized through the Design of Experiment (DoE) methodology. Finally, the physico-chemical properties and in vitro cytocompatibility on neonatal human dermal fibroblast cells of these constructs were evaluated. Additionally, the effect of membranes on the expression of healing-associated genes was assessed via RT-qPCR and their antibacterial efficacy against various bacterial strains, including *Staphylococcus aureus* and *Pseudomonas aeruginosa* was confirmed. For the first time, we aimed to demonstrate the potent synergy between Manuka honey, chitosan, and essential oils in combating bacterial infections.

Combining Manuka honey with essential oils enhances antimicrobial effectiveness, targeting both Gram-positive and Gram-negative bacteria. Manuka honey’s methylglyoxal primarily combats Gram-positive bacteria^28^, while TEO and CEO address a broader range, including antibiotic-resistant strains^29,30^. This innovative approach offers a holistic, sustainable solution to bacterial infections.

## Materials and methods

### Materials and chemicals

TEO and CEO were purchased from Holland & Barrett, UK. Tween20, Tween80, polycaprolactone (PCL; medium molecular weight MW =  ~ 80 kDa), hexamethylenediamine 98% and chitosan (low molecular weight, deacetylation degree 75%) were supplied by Merck, UK. Manuka Honey (MGO 550 + . Medical grade) was purchased from Manuka Health, USA. All solvents were of analytical grade and used with no further purification. They were all purchased from Merck, UK. Distilled water (dH_2_O) and LC/MS-grade water were obtained throughout PureA-Q + System (SLS-LabPro, UK).

### Manufacturing methods

#### Preparation of TEO and CEO nanoemulsions

The oil-in-water emulsions were prepared following the procedure outlined by Zhang et al.^31^. EOs was dispersed in dH_2_O at different concentrations, using Tween 80 as emulsifier at a weight ratio of 66.7% relative to EOs. The mixture was stirred at 600 rpm for 10 min using a magnetic hotplate (Ika, Germany). Subsequently, the mixed dispersion was homogenized with a T25 digital ULTRA-TURRAX® (Ika, Germany) for additional 10 min at different speed according to the DoE. Finally, the resulting nanoemulsion was sonicated in a Labsonic ultrasonic bath operating at 140W and 50 kHz (FALC Instruments, Italy). To optimize the production of the essential oil nanoemulsion for achieving the smallest size, we employed a DoE based on a Response Surface Methodology with a central composite design. The factors included the EOs concentration (% w/v), homogenization speed (rpm), and sonication time (min), while the resulting nanoemulsion size (nm), analyzed using dynamic light scattering (DLS), served as the response variable. The experimental setups were conducted using Minitab Statistical software (version 21.4 64-bit, https://www.minitab.com/en-us/). Table 1 outlines the ranges of the factors examined in the manufacturing of the EO nanoemulsion.

**Table 1:** Range of experimental variables for the response surface design applied in the essential oil nanoemulsion manufacturing.

	Factor	Level				
		− α	− 1	0	1	α
X_1_	Essential Oil concentration (% w/v)	0.734	2	4	6	7.266
X_2_	Homogenization speed (rpm)	5917.5	7500	10,000	12,500	14,082.5
X_3_	Sonication time (min)	1.5	4	8	12	14.5

#### Manufacturing of electrospun membranes

PCL pellets were dissolved in acetic acid and formic acid (1:1 v/v) to reach a concentration of 17.5% (w/v) under stirring overnight and then, processed through a Spinbox electrospinning (Bioinicia, Spain). The electrospinning parameters were fine-tuned for the process. Membranes were produced using a stationary 21G needle and a flat paper plate to gather fibers oriented in a random fashion. The solution was deemed suitable for spinning under the specified conditions: a distance of 12 cm from the tip to the metallic collector, a flow rate of 600 µl/h, and an electric potential of 16 kV. The resulting membranes were placed under a hood overnight to ensure the evaporation of any residual solvent.

#### Coating of the electrospun membranes via LbL assembly technique

**Scheme 1:**
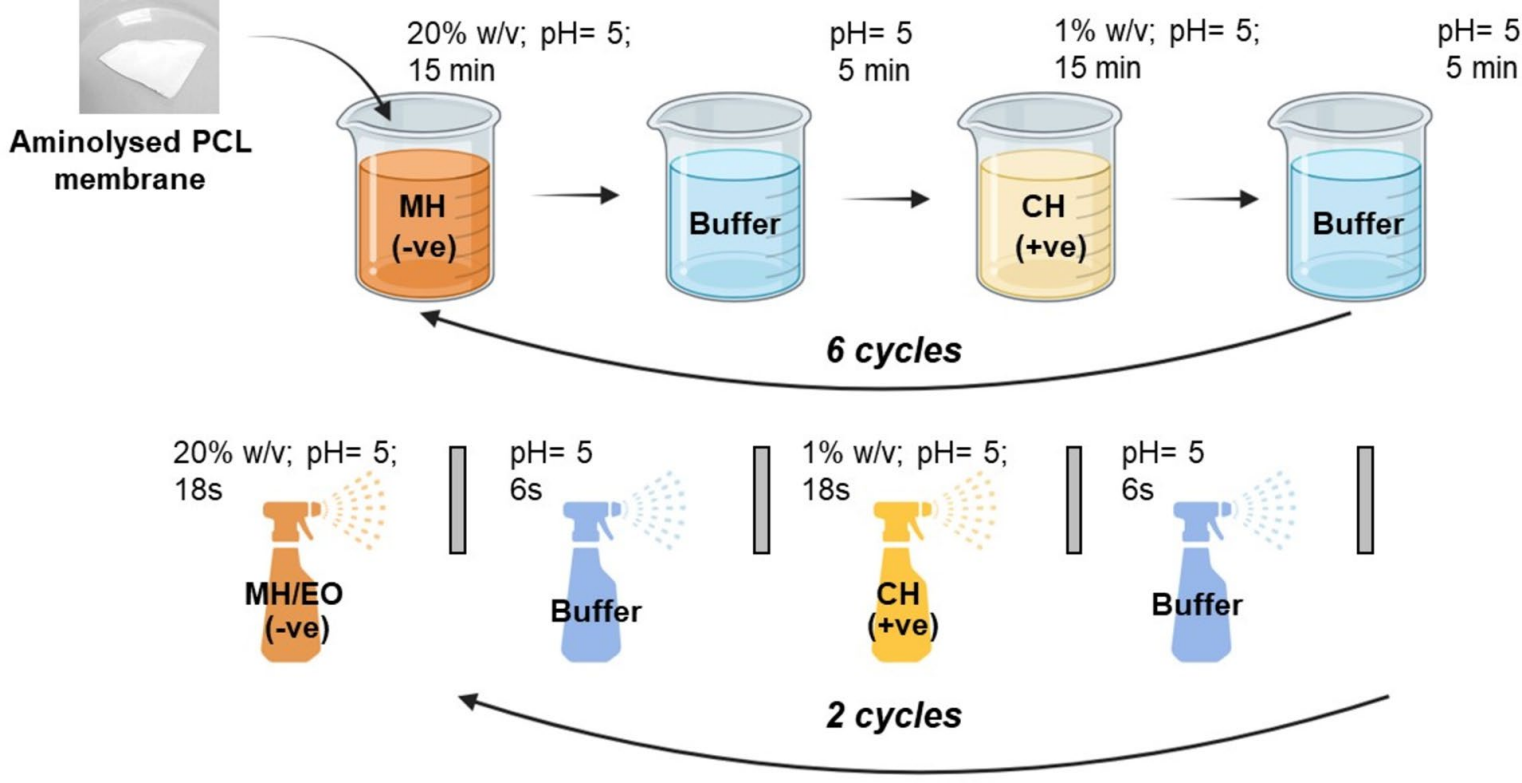
LbL functionalization of aminolysed PCL electrospun membranes that combines dipping (6 cycles) and spray (2 cycles) modality by using an automatic hybrid device developed at Newcastle University (WO 2021079106A1).

Prior to the LbL functionalization, the membranes were aminolyzed according to Mancuso et al.^28^. The electrospun membranes were immersed in an hexamethylenediamine (HED) solution (0.06 g/ml) for 15 min at 20 °C, aiming to graft -NH_2_- groups onto the surface to introduce a positive charge. Subsequently, the aminolyzed meshes were rinsed five times with dH_2_O and left to dry under a hood for 12 h. Solutions of Manuka Honey (20% w/v) and chitosan (1% w/v) were prepared dissolving them into sodium acetate buffer at pH 5. The LbL functionalization was performed using an automatic hybrid device developed at Newcastle University (WO 2021079106A1) that combines dipping and spray LbL assembly, as illustrated in Scheme 1. The membranes were dipped into the polyanionic MH solution for 15 min, followed with a washing step into sodium acetate buffer for 5 min to remove any unbound material; then, the polycation CH solution was used to form the second layer using the previously outlined method. The process of immersing the material in the polyelectrolyte solutions was reiterated until a total of 12 alternating charged layers had been successfully applied. Subsequently, an additional four layers were applied to the coating using a spraying technique to integrate the essential oil nanoemulsions, that were formulated based on the optimal parameters derived from the DoE analysis. The pH of these EO nanoemulsions was set to 5, and blended with the MH solution (with a ratio of 1:15 EO to MH), given their negative charge as verified by ζ-potential analysis. The application involved spraying both CH and MH/EO polyelectrolytes onto the membrane for a duration of 18 s, followed by a 6-s spray of washing step using the sodium acetate buffer. After completing the surface functionalization, the membranes were allowed to air-dry overnight and subsequently stored at 4 °C until further use.

### Physico-chemical characterization methods

#### Dynamic light scattering (DLS)

The size, polydispersity index (PDI) and ζ -potential value of the EO nanoemulsion were analyzed using a Litesizer 500 (Anton Paar, Austria) device. The set parameters were an equilibration time of 120 s, 15 runs at room temperature (25 °C) and scattering angle of 173°. All the measurements were repeated three times.

#### Transmission electron microscopy (TEM)

The EO nanoemulsion morphology was investigated through observation with TEM. A drop of the solutions was placed onto a copper grid and the excess of liquid was wiped with a paper filter. The samples were observed under a CM 100 Compustage FEI TEM (Philips, Netherlands), with HV = 100 kV and digital images were collected using an AMT CCD camera (Deben) with a range of magnification up to 130,000×.

#### Quartz crystal microbalance with dissipation (QCM-D)

QCM-D analysis was performed to simulate the formation of the LbL coating and checking the electrostatic interactions of the selected polyelectrolytes with and without incorporation of the EOs. The analysis was performed using Qsense Explorer instrument (Biolin Scientific, Sweden) equipped with a gold-coated quartz crystal sensor (QSX 301) and an open module. The aminolysis process was simulated pouring ED solution onto the sensor for 10 min followed by a washing step with sodium acetate buffer. The LbL coating was carried out as described before, alternatively pouring the polyelectrolyte solutions for 10 min, separated by 5 min washes with sodium acetate buffer, for 16 layers in total. Changes in frequency (Δf) and energy dissipation factor (ΔD) were monitored real time at its fundamental resonance frequency (5 MHz) and odd overtones (3, 5, 7, 9, 11, 13).

#### Scansion electron microscopy (SEM)

SEM analysis was initially employed to assess fiber diameters and the presence of defects in the manufacturing electrospun membranes. Subsequently, it was utilized to determine the morphology of the membranes after the LbL assembly functionalization. The analysis was conducted using the JSM-5600LV (Jeol, Japan) equipment. Prior to examination, the samples were coated with gold sputters (applied using a Sputter Coater from BIO-RAD Microscience Division). The captured images were subsequently processed and analyzed using ImageJ software (version 1.53, https://imagej.net/ij/).

#### Fourier transformed infrared spectroscopy with attenuated total reflectance (FTIR-ATR)

FTIR-ATR analysis was conducted to investigate the functional groups on the surface of the samples. Measurements were obtained with a Spectrum Two PE instrument equipped with a horizontal attenuated total reflectance (ATR) diamond crystal (PerkinElmer Inc., USA). Data were collected in absorbance mode, by averaging 32 scans, with wavenumber values ranging from 4000 to 550 cm^−1^ (resolution = 2 cm^−1^).

#### X-ray photoelectron spectroscopy (XPS)

XPS was employed to quantitatively measure the elemental composition of the first superficial layers of the material, as well as to determine the binding states between the elements (specifically C, N, O). The samples were examined by a scanning microprobe Kratos Axis Ultra-DLD XPS spectrometer (EPSRC Harwell XPS Service Cardiff, UK), equipped with a monochromatized AlKα X-ray radiation source. The base pressure in analysis chamber was 10^−9^ mbar. Samples were analysed in High Power mode with an X-ray take-off angle of 45° (scanned size∼1400 × 200 μm). For each specimen, survey scans (Fixed Analyser Transmission mode, binding energy range 0–1200 eV, pass energy 117.4 eV) and high-resolution spectra (FAT mode, pass energy 29.35 eV) were acquired. Atomic concentration (At.%) analysis on the survey scan was performed using the built-in CasaXPS software package (version 2.3.25PR1.0 http://www.casaxps.com/). Subsequently, to detect the binding energy related to the chemical binding states of each element within the films, the XPS spectra for the chemical elements detected from the films were subjected to peak deconvolution using the same software.

#### Determination of the MGO and EOs quantification and their in vitro release profile

LbL functionalized membranes with and without EO nanoemulsions were placed in a 12-well plate with 1 mL of dH_2_O and then incubated for 2 h at room temperature while shaking. Subsequently, an MGO assay kit based on calorimetry was used to assess the MGO concentration in the LbL coated samples. Briefly, 20 μL from the released solutions were added to a 96-well plate and 80 μL of the MGO assay kit solution (Abcam, UK) was introduced and allowed to incubate for 2 h at room temperature. The MGO content was then determined by measuring the absorbance values at 450 nm using a FLUOstar Omega microplate reader (BMG Labtech, Germany). Likewise, a standard curve for MGO was prepared by preparing MGO solutions ranging from 0 to 500 µM, combining them with the MGO assay kit, and then assessing their absorbance at 450 nm. Moreover, the released solution was analysed at 276 nm using a microplate reader to assess the content of essential oils incorporated into the nanolayers. Similarly, a standard curve for the EOs was established for both TEO and CEO by preparing solutions ranging from 0 to 250 µM and assessing their absorbance at 276 nm.

The stability of the LbL-functionalized samples and the release of MGO and EOs were examined at specific intervals (1, 2, 4, 8, 24 and 72 h, 7 and 14 days) in a phosphate-buffered saline (PBS) solution at 37 °C using MIDI 40 incubator (Thermo Scientific, UK). The quantification of MGO and EOs release was determined using the aforementioned assessment methods.

### In vitro biological evaluation

#### Cell culture and seeding

Neonatal Human Dermal Fibroblasts (NHDF) were sourced from Lonza Biosciences in Switzerland and cultured as per the supplier's guidelines. These fibroblasts were maintained at 37 °C in an environment containing 5% CO_2_. The culture medium used was Dulbecco's Modified Eagle Medium (DMEM, Merck, UK), supplemented with 10% fetal bovine serum (FBS), 2 mM l-glutamine, and a 1% antibiotic mixture of penicillin and streptomycin (100 U mL^−1^). Bare PCL and LbL = functionalized membranes, with and without incorporation of EOs, were fixed in cell crown inserts and placed into a 48 non-treated well plate. Before the cell seeding, the membranes were subjected to treatment with Sudan Black (SB, Merck, UK) to eliminate the autofluorescence (due to the presence of the natural compounds in the coating) from the electrospun membranes. A solution of 0.3% (w/v) SB was prepared using 70% ethanol. The membranes were submerged in this solution for 15 min, followed by three rinses in a PBS solution. Subsequently, they were sterilized under ultraviolet light for 30 min. Cells were seeded with a density of 5 × 10^4^ cells/well and cultured until the required time points. Cells seeded on polystyrene tissue culture plates were used as control.

##### Live/dead and PrestoBlue assays

To evaluate cell viability, the two-color fluorescence ReadyProbes™ Cell Viability Imaging Kit, Blue/Green (Thermo Fisher Scientific, UK) was performed 48 h after cell seeding. The assay was conducted according to the manufacturer's protocol. In brief, the L/D staining solution was created by adding 2 drops of each NucBlue® Live and NucGreen® Dead reagents in 1 mL of the full growth media. After the incubation, wells were twice rinsed with PBS and then exposed to 300 μL of the L/D staining solution for 30 min at 37 °C. Imaging was carried out using the EVOS M5000 fluorescence microscope (Thermo Fisher Scientific, UK).

For gauging the metabolic activity of Neonatal human-dermal fibroblasts, the PrestoBlue™ assay (Thermo Scientific, UK) was utilized. After 24-h, 48-h and 7-day incubation, the culture medium was aspirated, and samples were cleansed with PBS at 37 °C. The PrestoBlue reagent, also maintained at 37 °C, was diluted in DMEM at a 1:10 ratio. Subsequently, 200 μL of this solution was introduced to each well and incubated for an hour at 37 °C under 5% CO_2_. After the incubation, 100 μL from each well was transferred to a white-bottom 96-well plate, and fluorescence was gauged using a microplate reader (560 nm excitation and 590 nm emission). Results were normalized by subtracting the mean fluorescence of control wells that contained only the PrestoBlue solution. The data are represented as viability percentages, by normalizing the relative fluorescence units (RFU) detected for each sample against the control cells treated with DMEM only.

##### Gene expression analysis

The impact of membranes on the activation of genes related to tissue healing was assessed using RT-qPCR. Total RNA was isolated from the samples using the using the Trizol method (Invitrogen, UK), following the specified guidelines. The concentration and quality of the RNA obtained were determined using a NanoDrop™ 1,000 spectrophotometer (Thermo Fisher, UK). Subsequent reverse transcription was carried out using the High-Capacity cDNA Reverse Transcription Kit (Thermo Fisher, UK) and conducted in a thermocycler (Applied Biosystems, USA) with cycles set at 10 min at 25 °C, 120 min at 37 °C, and 5 min at 85 °C. For Reverse Transcription quantitative real-time Polymerase Chain Reaction (RT-qPCR), the TaqMan™ Fast Advanced Master Mix combined with TaqMan™ probes for FGF1, TGFβ1, VEGFA and COL1A1 (Thermo Fisher Scientific, UK) were utilized. The raw gene expression data were collected using the QuantStudio™ 3 Real-Time PCR System (Thermo Fisher Scientific, USA). To standardize the results, the expression levels of the target genes were normalized against the reference gene Glyceraldehyde-3-phosphate dehydrogenase (GAPDH), and expressed relative to controls using the 2^−ΔΔCt^ method as reported by Scalzone et al.^32^. The expression levels under control conditions for day 0 were normalized to a value of 1. Subsequent expression levels for day 7 and 21 were then depicted as fold-changes relative to these initial controls.

#### Bacterial tests

To evaluate the antibacterial properties of the LbL-coated membranes, antimicrobial sensitivity was assessed against *S. aureus* Rosenbach BAA-2313 (ATCC, UK) and *P. aeruginosa* strain DSMZ 19,880 (DSMZ, Germany). Overnight cultures were prepared by suspending a colony in ~ 10 ml of Brain Heart Infusion (BHI) broth NCM0016B (Neogen, UK) and incubating at 37 °C for approximately 20 h. Subsequently, cell densities were enumerated by direct plate colony counting on BHI agar NCM0080A (Neogen, UK). Samples cut in circular discs (~ 12 mm in diameter) were prepared for each functionalized membrane and placed in a 24-multiwell plate. They were then irradiated for 30 min on each side using ultraviolet light in a UV sterilizer Cabinet CL-1000 (Akribis Scientific Limited, UK). To assess bacterial inactivation, 200 μL of overnight culture of each bacterial species was inoculated separately onto each disc. For the XTT assay, 100 μL of overnight culture of each bacterial species was homogenized in 5.9 mL of MRD using a LP Vortex Mixer (Fisher Scientific, UK), and 200 μL of the resulting suspension was inoculated onto each disc. The multiwell plate was sealed with Parafilm (Merk, UK) and then incubated at 37 °C for 24 h. Coverslips (13 mm in diameter, Agar Scientific, UK) were utilized, onto which 200 μL of overnight bacterial culture was added, with bacteria treated with gentamicin (15 μg/mL, Merk, UK) serving as the positive control and untreated bacteria as the negative control.

##### Bacterial enumeration

After the incubation period, each membrane and coverslip were suspended in 5 mL of maximum recovery diluent (MRD) and sonicated at 60 Hz in an E30h ultrasonic bath (Elma, Germany) for 7.5 min to facilitate detachment of the bacteria from the membranes. Subsequently, serial dilutions were prepared in MRD, and 100 µL of each dilution were spread-plated on BHI Agar (Neogen, UK), and then incubated at 37 °C for 24 h to enumerate bacteria and calculate bacterial reduction on the membranes. Values from the enumeration of survivors in the overnight culture were used to cross-check the initial bacterial load inoculation with the bacterial reduction.

##### XTT assay

The XTT solution (CyQUANT XTT Cell Viability Assay, ThermoFisher Scientific, UK) was prepared according to the manufacturer's instructions. Briefly, 1 mL of Electron Coupling Reagent was added to 6 mL of XTT Reagent, vortexed to ensure thorough mixing, and 70 µL of the prepared solution was added to each well. The well plates were then incubated at 37 °C for 4 h. Following the incubation period, the absorbance at 450 and 660 nm was measured using the FLUOstar Omega microplate reader. The tests were conducted in triplicate.

##### Bacterial growth assessment by live/dead staining

Electrospun membranes were exposed to 200 µL of growth medium containing 10^6^ of *S. aureus* and *P. aeruginosa*, then incubated at 37 °C for 24 h. Subsequently, they were washed thrice with PBS. Dyes were prepared by combining 1.5 μL of Syto-9 and 1.5 μL of propidium iodide (PI) reagents (LIVE/DEAD BacLightTM Bacterial Viability Kit, ThermoFisher Scientific, UK) with 1 mL of PBS. The samples were then treated with 0.5 mL of the Syto-9 and PI mixture and left in the dark for 15 min, followed by two rinses with PBS. Finally, the membranes were placed upside down on microscope coverslip and examined under a confocal laser scanning microscope (Leica TCS SPE, UK).

### Statistical analysis

The results were presented as means ± standard deviations. Initially, a One-way ANOVA with repeated measurements was employed. Subsequently, a Tukey's post hoc test was conducted to identify the main factors contributing to data variability. The level of statistical significance was set as follows: ∗ for *p* < 0.05, ∗  ∗ for *p* < 0.01 and ∗  ∗  ∗ for *p* < 0.0001. Statistical analysis was carried out using GraphPad Prism Software (version 8.4.1 https://www.graphpad.com/).

## Results and discussion

### Optimization of the manufacturing of the EOs nanoemulsions

EOs are naturally occurring aromatic compounds known for their broad range of biological effects^33^. Over time, they have been utilized for various purposes, including as flavor enhancers, therapeutic agents in medicines or cosmetics, and for their insecticidal, antioxidant, anti-inflammatory, anti-allergic, and anticancer properties^34^. Notably, many EOs display potent antibacterial, antiviral, and antifungal properties, leading to their use as natural antimicrobial agents^35^. To maintain their biological efficacy, EOs encapsulated nanoemulsions present different benefits, including enhanced bioactivity due to their small size and improved diffusion properties. Furthermore, the surface-active properties of surfactants and emulsifiers in nanoemulsions can further enhance their antimicrobial and anti-biofilm effects^36^. However, to date, works supporting the utilization of nanoemulsions containing antimicrobial EOs within multilayered nanocoating in biomedical applications have not been reported.

**Fig. 1:**
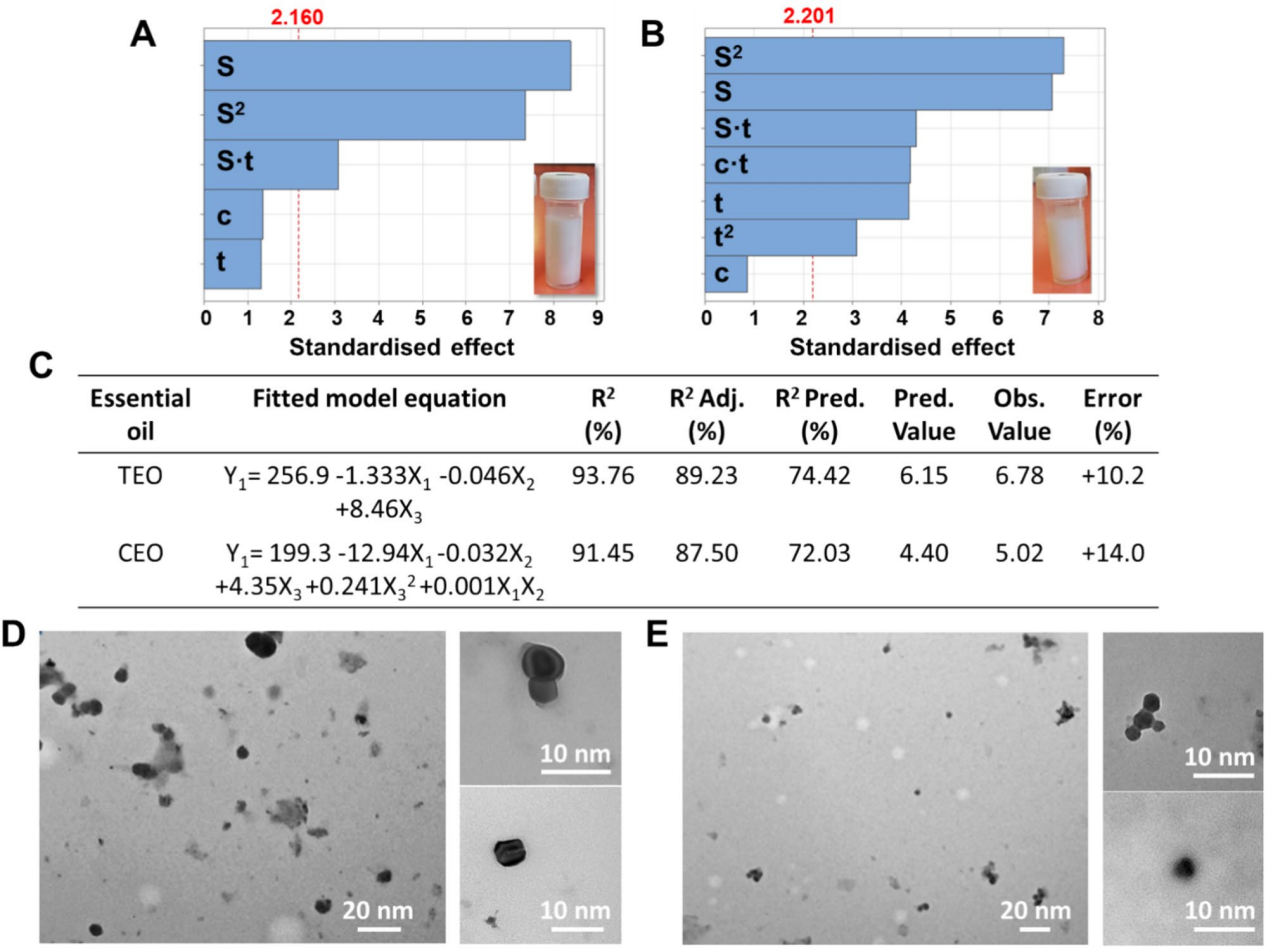
Pareto charts of the standardized effects of the factors (c: EO concentration, S: Homogeniation speed, t: Sonication time and their interactions on the response variable (α = 0.05) of the resulting size of the TEO (**A**) and CEO (**B**) nanoemulsions, along with (**C**) fitted model equations and metrics for each procedure. TEM images of TEO (**D**) and CEO (**E**) nanoemulsion.

In this study, various extraction methods were employed and evaluated to determine the optimal procedure for producing essential oil nanoemulsions with a size suitable for incorporation into multilayered nanocoatings. Given that the typical thickness of the resulting LbL nanolayers ranges from a few to 30–50 nm, we identified the optimal size range for the nanoemulsion to be between 1 and 20 nm, that can be achieved following Zhang’s procedure^31^, as response variable of the DoE. Figure 1 A–C display the factors (EO concentration, homogenization speed, and sonication time) and their interactions that most influence the evaluated processes. It was observed that the homogenization speed (indicated with S) significantly influenced the manufacturing of both EOs, while the sonication time (t) significantly influenced the size of the cinnamon EO nanoemulsion, resulting in a smaller size range compared with the TEO. Notably for both nanoemulsions, the optimized conditions for the oil concentration and sonication time reached the upper limit of the factors (X_1_ and X_3_) in the DoE, while the optimized values for the homogenization speed was found as 12,515 rpm and 13,635 rpm for the CEO and TEO respectively (Table S1). As observed in our work, the EO concentration did not influenced significantly the nanoemulsion size. As reported in literature, the weight ratio between the EO and surfactant could affect the resulting size. Zhang et al.^37^ found that an increase of the surfactant content (from an oil-surfactant ration of 3:7 to 1:9) can decrease the particle size from 28 to 8 nm. This is because the primary function of surfactants is to stabilise the emulsion by lowering the surface tension at the oil/water interface^38^.

For both TEO and CEO, the R^2^ coefficients were above 90% and the prediction coefficients (R^2^ Pred) over 70%, which may be attributed to the simplicity of the manufacturing process, indicating a strong interaction between the response and independent variables^39^. The percent error for this extraction, calculated based on the predicted and observed values, was around 10%.

Then, the PDI is not utilized as a response variable in the DoE. Instead, it served to assess the uniformity of droplet size distribution, reflecting the range of particle sizes present in each sample. PDI values range from 0 to 1, with a value below 0.3 generally considered indicative of a homogeneous sample^40^. In our work, the PDI remained below 0.3, confirming that the samples were monodispersed, and each portion was representative of the entire sample (Table S2).

Moreover, using the optimized conditions indicated by the DoE (Table S1), the ζ-potential values of the emulsions was assessed using the DLS, detecting − 16.9 ± 1.8 mV for the TEO nanoemulsion and − 21.6 ± 2.2 mV for the CEO nanoemulsion. This suggests that, during the LbL assembly, the essential oil nanoemulsions should be incorporated into the polyanionic solution—in this case, the one containing Manuka honey. Furthermore, ζ-potential measurements can indicate emulsion stability and propensity for particle aggregation: higher absolute values typically signify stable particles and minimal agglomeration. According to literature, ζ-potential values exceeding 20–25 mV (in absolute terms) suggest that repulsive forces among particles surpass attractive forces, ensuring particle dispersion^28^. This is in accordance with our manufactured nanoemulsions characterized by a PDI of ~ 25% as discussed before.

Finally, TEM analysis was conducted to morphologically characterize the EO nanoemulsions in terms of their shape and size (Fig. 1 D-E). Both droplet particles exhibited a spherical shape, with varying sizes and agglomeration observed. The average particle diameter was determined in the range from 2 to 10 nm for both TEO and CEO.

### Physico-chemical characterization of the LbL-functionalized membranes

In this work, the LbL assembly process was used to functionalize the surface of electrospun PCL membranes by using CH and MH (with and without the incorporation of the manufactured EO nanoemulsions) as polyelectrolytes (PEs). Electrophoresis measurements revealed that MH-based solutions carried negative charges with ζ-potentials between − 22.4 ± 3.4 mV without EOs, and − 19.7 ± 1.8 mV and − 21.6 ± 2.2 mV with TEO and CEO respectively. In contrast, the CH solution displayed a positive charge with a ζ-potential of + 36.5 ± 2.4 mV. These findings are consistent with literature, suggesting that both MH and CH can be utilized in the fabrication of multilayered coatings through LbL. Indeed, their use has been combined with other polyelectrolytes, such as the polycation polyallylamine hydrochloride (PAH) and the polyanionic pectin has been reported in previous works^28,41^.

To validate the formation of multilayered structures, the electrostatic interaction between MH and CH was explored using QCM-D measurements^42^.

**Fig. 2:**
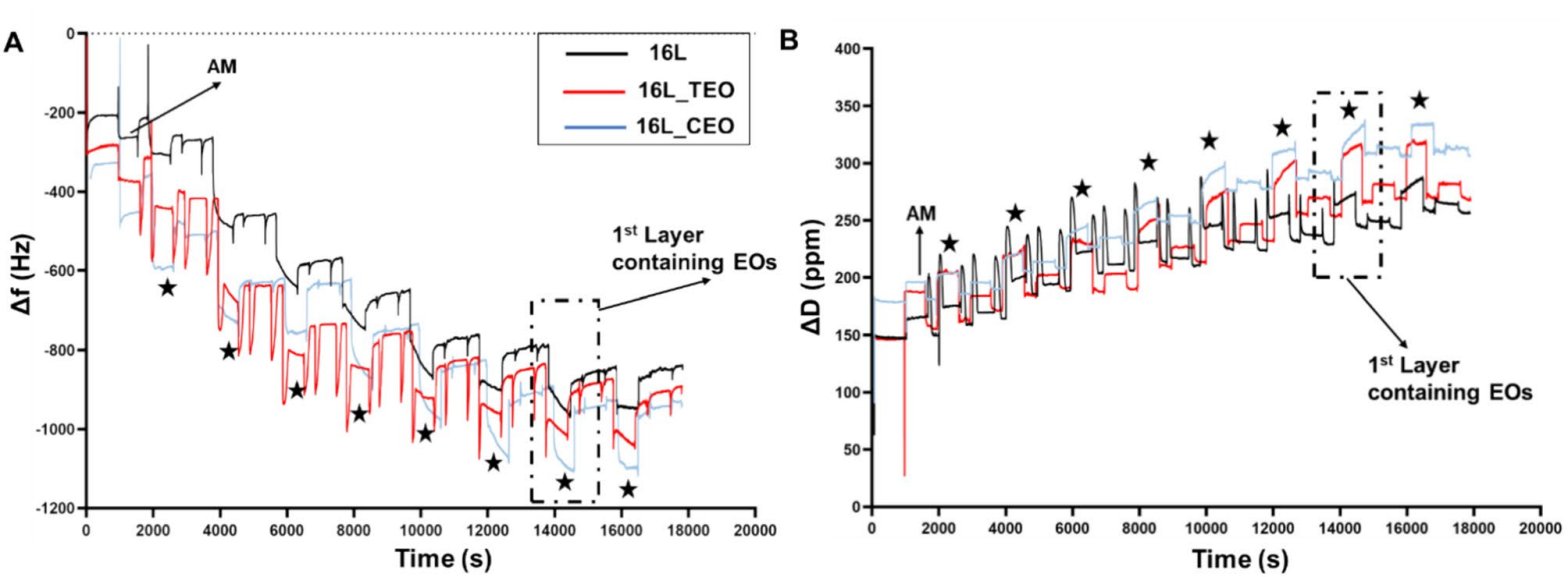
QCM-D analysis to evaluate the electrostatic interaction of the selected polyelectrolytes for the LbL assembly functionalization: frequency shifts (**A**) and dissipation shifts (**B**) of the fifth overtone versus time (s).

Figure 2 illustrates that the introduction of the ED solution (labelled with AM) induced alterations in both frequency and dissipation as NH_2_ groups engaged with the gold surface of the sensor. A subsequent cleaning phase partially eradicated this layer, as evidenced by the Δf value increase (Δf value post-cleaning ≈ − 350 Hz) around 600 s. Following this, MH and CH polyelectrolyte solutions were sequentially passed over the positively charged Au crystal for 10 min, mimicking the LbL dipping conditions of electrospun membranes. The deposition of PEs molecules onto the crystal surface was evident through a progressive, stepwise response in both Δf and ΔD values, with the black stars denoting shifts attributed to MH-based solutions. By already the second bilayer, the shifts caused by the Manuka Honey solution were broader than those resulting from the introduction of the chitosan solution, indicating a greater mass contribution from the MH layer. Additionally, the ΔD trajectory signified the accumulation of a denser layer atop the Au crystal, as the dissipation factor escalated over time. Furthermore, the incorporation of the EOs nanoemulsion did not affect the electrostatic interaction between the PEs. However, the sample containing TEO exhibited reduced slopes in both frequency and dissipation respect with CEO.

Biomimetic fiber-based membranes were created using electrospinning to replicate the nanofibrous architecture of the native ECM^43^. In this work we selected the PCL due to its recognized biocompatibility and its ability to maintain stability during LbL processing without compromising its inherent properties or structural morphology. The membranes produced were entirely free of defects as shown in Figure S1. The fibers exhibited nanoscale dimensions, with a mean diameter of 211 ± 72 nm and displayed a uniform distribution.

**Fig. 3:**
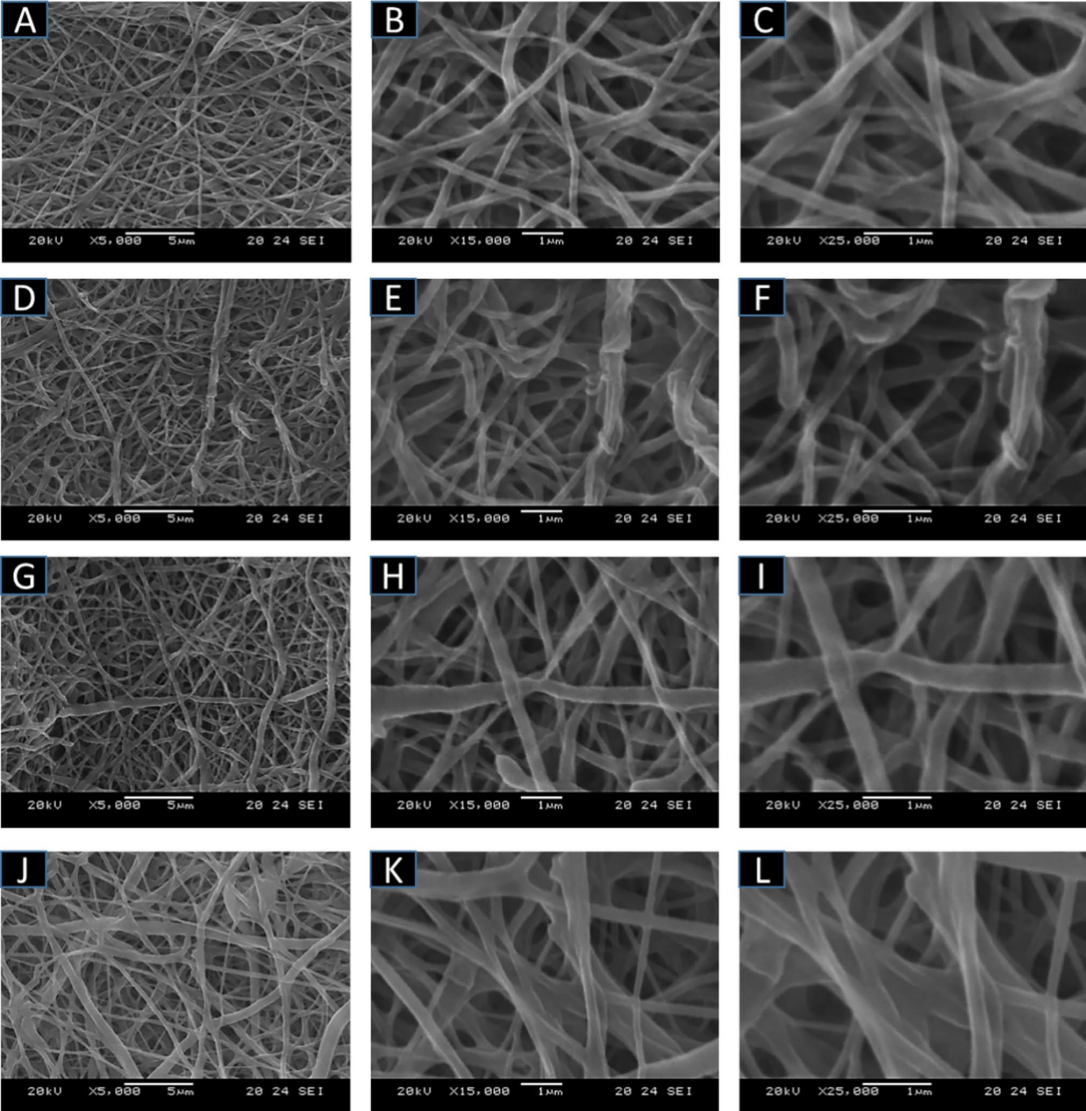
SEM images of the LbL-coated electrospun membranes at different magnifications (from left to right: X5000, X15000, X25000) with 8 layers (**A-C**); 16 layers w/o essential oils (**D-F**); 16 layers with incorporation of tea tree EO nanoemulsions (**G-I**); 16 layers with incorporation of cinnamon EO nanoemulsions (**J-L**).

For the optimal functionalization of PCL-based electrospun membranes via LbL assembly, the parameters were established following previous research of the authors^28,44^, that include 20% (w/v) of MH and 1% (w/v) CH concentrations respectively, with 15 min of dipping time to get 16 nanolayers. The surface structure of the multilayered coating post-LbL assembly was examined using scanning electron microscopy (Fig. 3).

To enhance the initial PE absorption, an aminolysis treatment was conducted prior to LbL assembly, introducing primary and secondary NH_2_ groups onto the substrate^45^. This surface modification preserved the membranes' inherent microporosity, crucial for cellular penetration, nutrient diffusion, and waste removal, and ensured they presented biocues akin to the native ECM^43^.

Specifically, SEM analysis revealed that with the increase of the number of layers (Fig. 3 A-F) resulted in an increased surface roughness and fiber diameter compared to the uncoated substrate (Figure S1). This is common of the complexation between PEs, becoming more pronounced with an increased number of bilayers, as evidenced by multiple studies^46^. With the incorporation of the EOs within the nanolayers, the membranes were characterized by the presence of more agglomerates and more pronounced inhomogeneity of the fibers (Fig. 3 G–L). For membranes with an eight-layer coating, the mean diameter measured 289 ± 60 nm, while the sixteen-layer assembly recorded 374 ± 62 nm. These findings confirmed a linear trend between the number of layers and the thickness of the coated fibers. Consequently, the thickness of a single bilayer can be deduced mathematically, approximating it to 20 nm. The addition of sprayed-EOs nanoemulsions presented different outcomes of the average fiber diameter compared with the 16-layered coated membranes with CH and MH only. We observed similar fiber diameter for the TEO-loaded samples (380 ± 89 nm) while the CEO increases the diameter reaching an average value of 423 ± 74 nm. Despite this trend, these values can not be considered statistically different (p > 0.05). This tendency was in accordance with the QCM results. Furthermore, all the SEM images indicated that the nanocoating did not compromise the membrane's morphological characteristics.

**Fig. 4:**
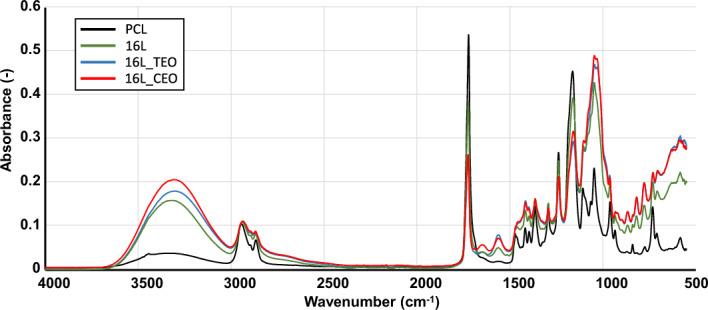
FTIR-ATR spectra of the electrospun membranes before and after LbL-functionalization. Inserts: magnification of the wavelength range where the main shifts or changes in peaks were observed.

Figure 4 shows the FTIR-ATR spectra of the surfaces of the electrospun membranes before and after the LbL assembly**.** The samples tested included aminolyzed PCL, a 16-layer coating without and with TEO and CEO nanoemulsions. The spectrum of the uncoated membrane exhibited characteristic wavelength bands of pure PCL with a pronounced peak at 1727 cm⁻^1^ that corresponded to the carbonyl stretching *v*(C = O) in the polymer, while peaks at 2866 cm⁻^1^ and 2945 cm⁻^1^ were attributed to the symmetric and asymmetric stretching of CH_2_ bonds, respectively^47^. Additionally, peaks at 1240 cm⁻^1^ and 1170 cm⁻^1^ corresponded to the asymmetric and symmetric stretching of C–O–C and O-C-O bonds, respectively. These PCL-related peaks were also evident in all the coated samples. Furthermore, the peak at 3460 cm⁻^1^ highlighted in the Figure 4, indicated the N–H stretching of amines in the uncoated sample^48^. This was consistent with the membranes being aminolysed using hexamethylenediamine to expose NH_2_ groups for the LbL process. For the 16-layer coated samples without EOs, new peaks emerged in the 1700–1500 cm⁻^1^ range. Specifically, peaks at 1690 cm⁻^1^ and 1641 cm⁻^1^ indicated the presence of Manuka honey, while a peak around 1570 cm⁻^1^ suggested the presence of chitosan^28,49^. Moreover, the sample incorporating CEO and TEO in the 16-layer coating exhibited no substantial differences between them. CEO and TEO's characteristic 'fingerprint' lies between 1800 and 600 cm⁻^1^ ^49^. However, its identification was challenging due to overlaps with other compound peaks, including peaks at 1727 cm⁻^1^ and around 1570 cm⁻^1^ intensified due to overlaps with aldehyde and C = C stretching of aromatic molecules and the broad peak between 3600 and 3000 cm^⁻1^, centered at 3300 cm^⁻1^, indicating the presence of OH groups in both polyelectrolytes and phenolic OH groups in the EOs^50^.

**Fig. 5:**
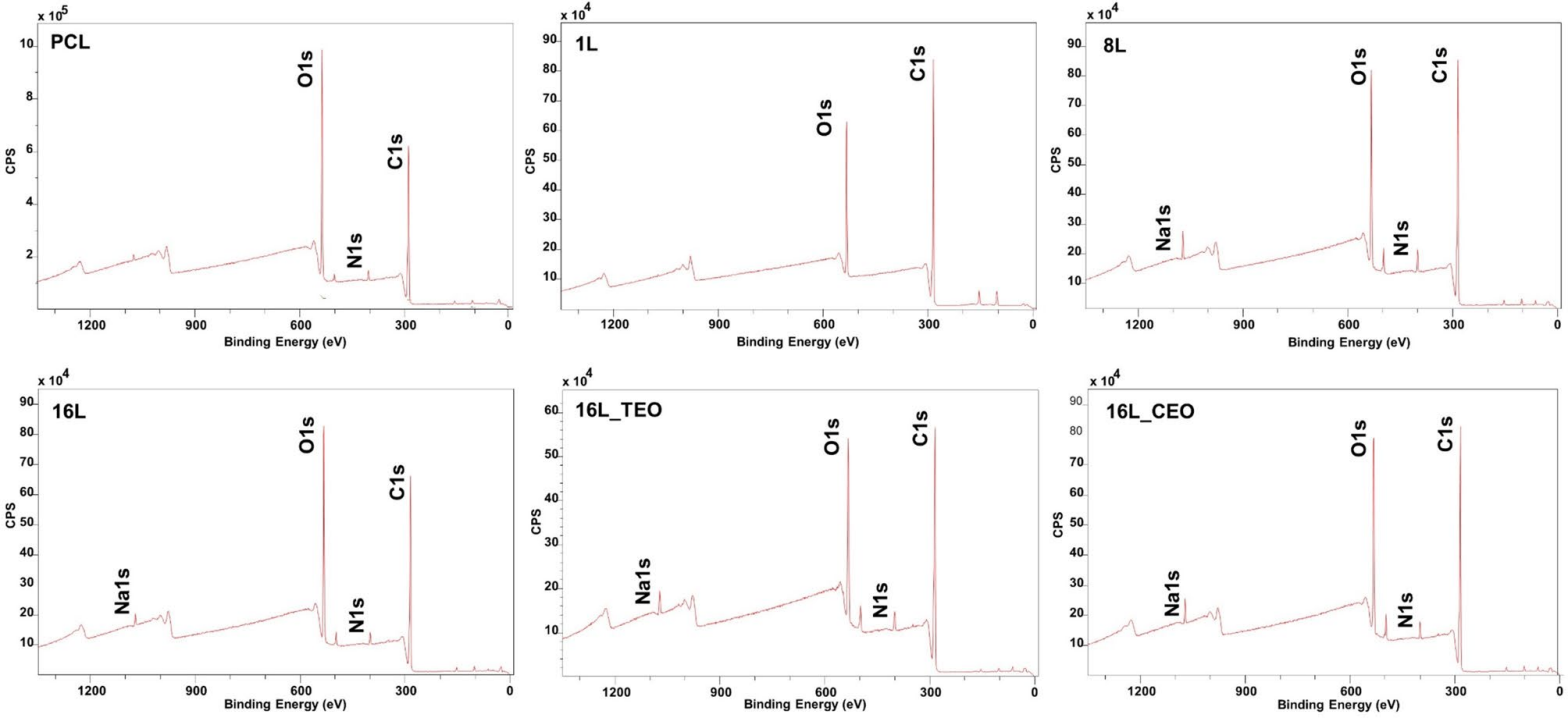
XPS survey spectra of the analyzed samples: aminolysed PCL electrospun membrane and after LbL functionalization with 1L, 8L and 16L without and with incorporation of the TEO and CEO.

The chemical composition of the membrane surfaces was confirmed using XPS analysis. Figure 5 presents the survey spectra of the samples, while Table 2 summarizes the atomic percentages of the detected elements. Carbon (C 1 s) at 285 eV and oxygen (O 1 s) at 532 eV were consistently found across all samples. Additionally, the uncoated PCL sample exhibited nitrogen (N1s) at 399 eV, attributed to the aminolysis process used for pre-charging positively the electrospun membranes. However, this nitrogen signal was absent in the samples with one layer, suggesting efficient interaction between the substrate and the first layer of MH, which lacks nitrogen atoms. The subsequent appearance of a strong nitrogen peak in the all the remaining LbL-coated samples indicated the presence of the chitosan, the positively charged polyelectrolyte, used for the building-up of the multilayered coating. Furthermore, sodium (Na1s), a major component of the buffer used for polyelectrolyte solubilization, was detected at 1070 eV. A minor silicon (Si2p) peak was observed at 102 eV, predominantly in the one-layer sample, that is associated with the tape used for fixing the samples prior to the analysis. No substantial differences were detected when TEO and CEO have been introduced, composed of C and O elements.

**Table 2:** Atomic concentration (%) of the characteristic elements present in the multilayered coating and the core-levels of electrospun membranes after LbL assembly.

Sample	Atomic concentration (%)				− C–H–/–C–C–	− C–O–	–C = O–	HO–C = O
	O1s	C1s	N1s	Na1s				
Aminolysed PCL	28.57	68.05	1.63	n.d	45.21	41.24	13.54	–
1L	18.57	74.83	n.d	n.d	76.52	15.54	–	7.94
8L	22.26	72.1	3.18	1.1	58.21	25.13	6.63	10.02
16L	26.19	69.84	1.85	0.68	56.32	29.47	9.68	4.53
16L_TEO	21.23	73.79	2.79	1.45	42.18	32.67	22.29	2.85
16L_CEO	22.77	72.48	2.17	1.26	57.12	22.19	15.52	5.17

**Fig. 6:**
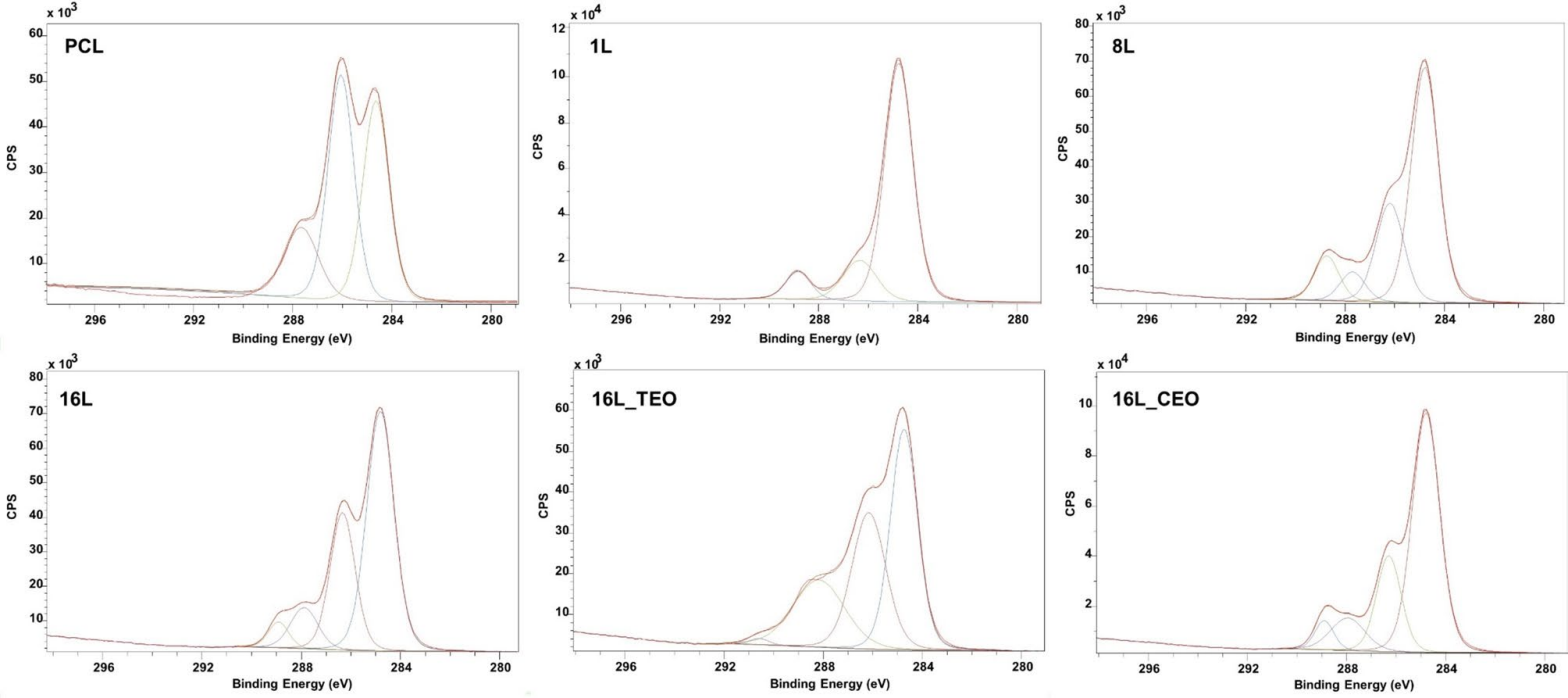
XPS high resolution C1s spectra of the analyzed samples: aminolyzed PCL electrospun membrane and after LbL functionalization with 1L, 8L and 16L without and with incorporation of the TEO and CEO.

In addition, high-resolution XPS spectra on C1s was performed, followed by spectral deconvolution (Fig. 6). Table 2 reported the detected atomic concentration of the bonds. For the aminolyzed PCL sample, the deconvolution of the C1s spectrum revealed peaks at 288 eV, corresponding to C–N bonds in addition to the typical characteristic PCL peaks at ~ 285 eV and 287 eV associated with the –C–H–/–C–C– and –C/O bonds respectively. With the 1-layer coated membrane, a peak was observed at ~ 289 eV, associated with the carboxylic group of the Manuka honey^51^. Interestingly, a decrease of the –C–O– bond was observed, compared with the aminolyzed samples (from 41 to 15%), due to the higher content of the peaks associated with –C–H– and –C–C– bonds (from 45 to 76%), typical of the Manuka honey. With the manufacturing of the 16-layered coating, both peaks at ~ 288 and 289 eV were detected corresponding to the bonds –C = O– and HO–C = O of Manuka honey and chitosan respectively. Electrostatic interactions between layers were further supported by specific bonding patterns (C–N–C and N–C = O). Moreover, changes in the atomic concentration of the bonds peak in the samples 16L_TEO and 16L_CEO were detected, confirming the incorporation of the EOs nanoemulsions.

**Fig. 7:**
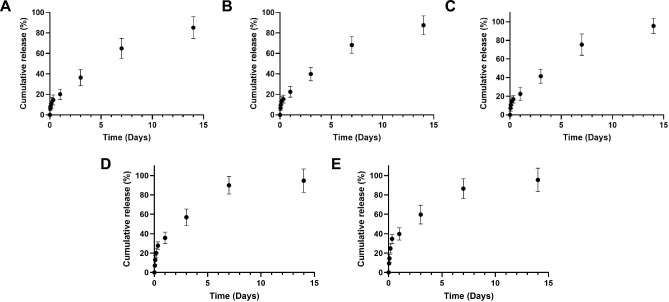
In vitro release profile at different time points of: (i) MGO from LbL coated membranes without EOs (**A**) and with incorporation of TEO (**B**) and CEO (**C**) and (ii) each EO from the membranes incorporating TEO (**D**) and CEO (**E**) respectively (n = 3).

The colorimetric MGO assay kit and the UV spectrophotometry were employed to determine both MGO and EOs concentration into the multilayered coating. Specifically, there is considerable interest in evaluating the MGO content and monitoring its release. Such monitoring is vital, since the release of MGO is expected to be closely linked to the therapeutic efficacy of these products, given MGO's essential role in the antibacterial attributes of non-peroxide honey^52^. Using an MGO standard curve (data not shown), we determined the MGO concentration to be approximately 18.2 ± 2.5 mM. This finding aligns with recent work by Udduttulla et al.^53^ wherein they functionalized Ti alloy substrates via LbL using a combination of hyaluronic acid and jellyfish-derived collagen. In their study, MGO was incorporated into the polyanionic solutions, resulting in concentrations ranging from 6.60 to 7.82 mM. This range is lower than the values observed in our study, likely because the antibacterial agent was incorporated only into the last three bilayers of the coating. Furthermore, the content of both EOs nanoemulsions was assessed ~ 1276 ± 165 µM for TEO and ~ 916 ± 212 µM for CEO. The literature discusses the incorporation of EO nanoemulsions into films. For instance, Elshamy et al. recently incorporated thyme EO nanoemulsions into chitosan films, achieving an estimated concentration of up to 0.4 mg/cm^3^ ^54^. The lower concentration of EOs in our study can be attributed to our method of incorporating the nanoemulsions into the nanocoating rather than into the structure of the electrospun membrane. Then, MGO and EOs release was monitored over 14 days as shown in Fig. 7. The LbL-functionalized membranes exhibited three distinct phases in their release profiles across all samples.

Considering the MGO release (Fig. 7 A–C), an initial burst release, accounting for ~ 15–20%, was evident within the first 24 h of incubation. Subsequently, a consistent and linear release of MGO persisted up to the 7 days, reaching a release in the range 65–75%. By 2 weeks, the release of MGO reached a value of 80%, with a remaining amount entrapped into the multilayered coating; that is expected to be released in the days following, due to the degradation of the nanocoating. This phenomenon was also observed in a previous work combining Manuka honey with the PAH to improve the antibacterial propertied of PCL electrospun membranes proposed to regenerate soft tissue^28^. The presence of the EOs did not influence the release of the MGO. Moreover, Fig. 7 D-E illustrates the sustained release of each EO over multiple days, displaying a similar triphasic release profile but characterized by a more pronounced initial burst that accounted ~ 40% within the first 25 h, while the subsequent 7-day period contributed to a cumulative release of ~ 90% for both samples. By 2 weeks, a slight increase of each EO release was detected, indicating the essential oils nanoemulsions were released within the 1 week of incubation. This observation aligns with the findings of Sotelo-Boyas et al.^55^. The elevated content of EOs observed in the PBS solutions within a short time frame may be due to the swift dissolution of EO molecules that were previously adsorbed onto the polymeric nanolayers. This facilitates their release into the surrounding environment. To mitigate the rapid release of EOs, as suggested by Ma et al.^56^, a viable approach could be the incorporation of nanoparticles that encapsulate essential oil molecules within their core. This encapsulation method can effectively minimise the loss, volatilization, and migration rate of essential oils into the external environment.

### In vitro biological characterization of the LbL-functionalized membranes

**Fig. 8:**
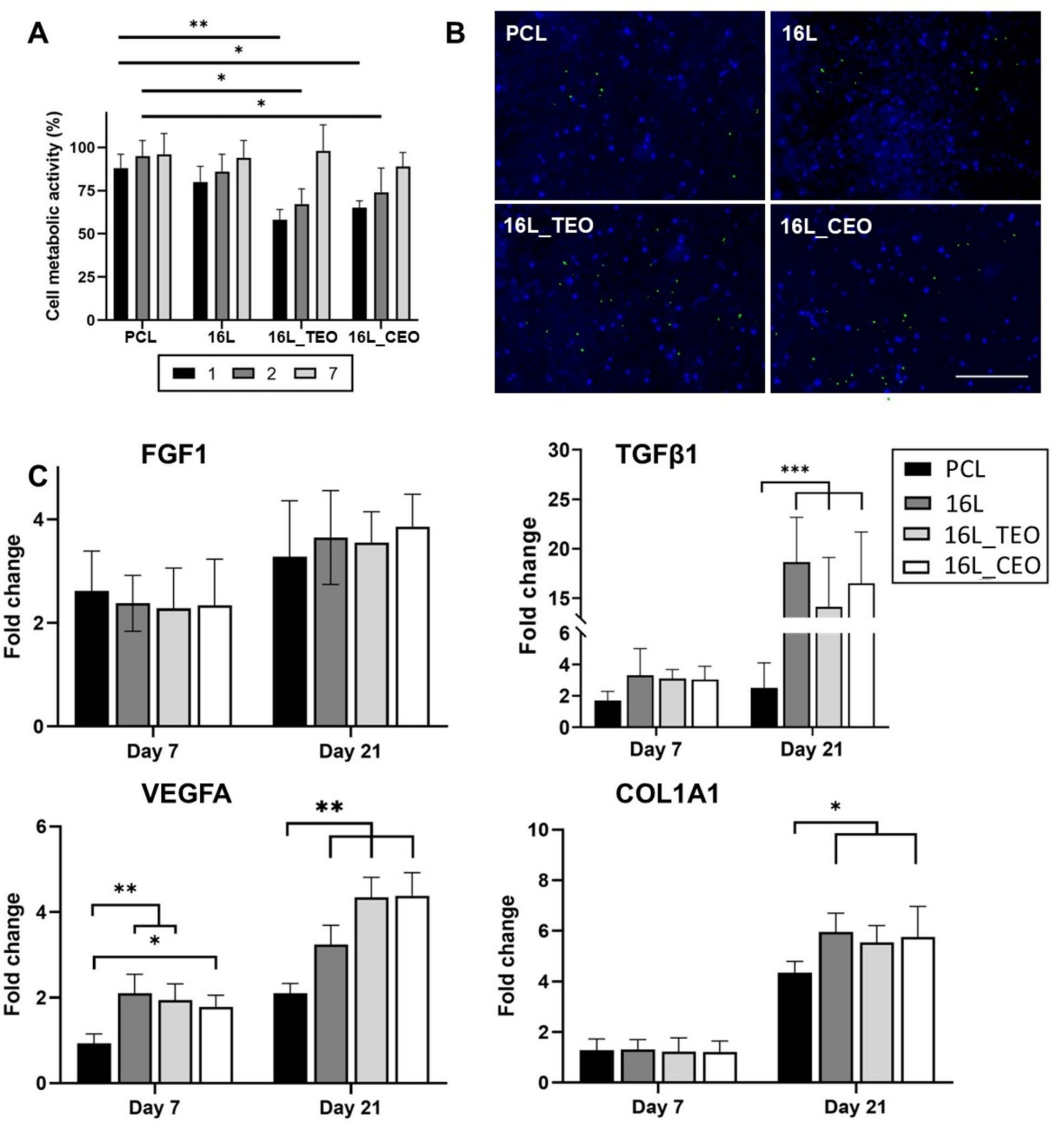
Metabolic activity of neo-dermal fibroblast cells seeded on electrospun membranes before and after LbL-functionalization. The results are shown as average ± SD after normalization to the control of cells seeded on TCPs (n = 3) (**A**); Live/dead images of Neo-dermal fibroblast cells after 48 h of seeding on electrospun membranes before and after LbL-functionalization. Scale bar = 300 μm (**B**). Gene expression analysis via RT-qPCR after 7 and 21 days of cells seeding on the electrospun membranes before and after LbL-functionalization (n = 5). The expression levels under control conditions for day 0 were normalized to a value of 1. Subsequent expression levels for day 7 and day 21 were then depicted as fold-changes relative to these initial controls. Statistics: ***p < 0.001, **p < 0.01, *p < 0.05 (**C**).

To evaluate the cytocompatibility of the LbL-functionalized membranes, we employed neonatal human dermal fibroblasts and their interactions with the samples is shown in Fig. 8.

The cell metabolic activity was assessed using the PrestoBlue assay against all the manufactured substrates, including the tissue culture plate (TCP) as control, over 1, 2, and 7-day period (Fig. 8A). The PrestoBlue findings indicated a significant increase in cellular metabolic activity throughout the incubation period for all tested samples. This can be attributed to the morphology of the electrospun membrane, specifically the fiber and pore sizes, which allowed cells to adhere and proliferate. Furthermore, interestingly, cells cultured on membranes containing EOs nanoemulsions exhibited reduced metabolic activity compared to the PCL substrate in the initial days of incubation (16L_TEO at 58 ± 6% (*p* < 0.01) and 16L_CEO at 65 ± 4% (*p* < 0.05) versus PCL at 88 ± 8% on day 1). The observed impact on cellular metabolic activity may stem from the antiproliferative effects of the EOs when released in elevated concentrations. Abd-Rabou noted that nanoemulsions of *Nigella sativa*-EO inhibited the proliferation of hepatocellular carcinoma cells more effectively than pure EO after a 24-h incubation. Specifically, these nanoemulsions demonstrated inhibition rates of ~ 29% and ~ 15% against HepG2 and Huh-7 cells, respectively^57^. Such antiproliferative effects are likely linked to the presence of terpinen-4-ol^58^ and cinnamaldehyde^59^, key chemical components found in TEO and CEO. Notably, the LbL- functionalized membranes showcased no significant variance compared to the PCL samples for each time point (*p* > 0.05). These findings affirm that the incorporation of MGO into the HA/J-COLL multilayered coating did not compromise the metabolic activity of L929 fibroblasts, underscoring the cytocompatibility of MGO-infused multilayers.

Additionally, a qualitative evaluation of fibroblast interactions with the samples was performed using fluorescence-based live/dead assays after 48 h of incubation (Fig. 8B). All samples displayed limited cell coverage, as evidenced by the blue staining. Both PCL and LbL-functionalized membranes without EOs showed a similar behavior to the cells seeded on Tissue Culture Plates (TCP) (Figure S2), but with a higher cell count compared to the 16L_TEO and 16L_CEO samples. The predominant blue staining of the cells suggested a largely viable cell population, with very few or no green-stained cells, indicating minimal cell death. These findings align with the metabolic activity results, reinforcing the nanolayers' ability to maintain optimal cell viability.

The LbL-functionalized membranes' intrinsic ability to foster new ECM formation was evaluated through gene expression analyses (Fig. 8C). The mRNA content of *FGF1* remained stable across all samples at each time point, with a slight increase noted by day 21. RT-PCR results indicated that the coated samples significantly enhanced the expression of VEGF, COL1, and TGF-β1 in the healing tissue, facilitating angiogenesis and tissue regeneration respectively^60,61^. Specifically, *TGFβ1* mRNA levels were detectable by day 7 and showed a marked increase by day 21 in the LbL samples (*p* < 0.001). In contrast, *COL1A1* exhibited significant upregulation only by day 21 (*p* < 0.001). Additionally, VEGF gene expression was noticeable by day 7 and experienced a substantial boost by day 21 (*p* < 0.01). Importantly, the inclusion of EOs nanoemulsions positively influenced the upregulation of these genes, particularly enhancing *VEGFA* levels. These findings align with literature reports where PCL/gelatin electrospun membranes, enriched with lawsone from *Lawsonia inermis* L. (Henna), exhibited a notable increase in TGF-β1 and COL1 gene expression after seeding with normal human gingival fibroblasts for 48 h^62^. Moreover, research on a herbal combination comprising *Aloe vera*, *Commiphora molmol*, *Adiantum capillus-veneris*, and henna has highlighted the upregulation of *TGF-β1* and *VEGFA* genes in mouse dermal fibroblast cells^63^. Notably, this study emphasized that the ethanolic extract of henna notably amplified this gene expression compared to other herbal extracts.

### Inactivation of bacterial species on LbL-functionalized membranes

Bacterial pathogens present a challenge to wound healing, when these organisms invade associated skin and soft tissue; such infections can range in severity from relatively mild sequalae through to life-threatening infection^64^. Commonly associated pathogens include Gram-positive species such as *S. aureus* and streptococci, although Gram negative species are also prominent in such infections, with *P. aeruginosa* a frequently implicated species^65^. The differing composition of the cell wall of Gram-positive and negative species is associated with differences in efficacy of various antimicrobial agents against these groups. The interaction of two bacterial species, *S. aureus* and *P. aeruginosa* (Gram-positive and negative species, respectively) with the LbL membranes generated in this study were examined, to understand the range of activity of different membrane compositions.

**Fig. 9:**
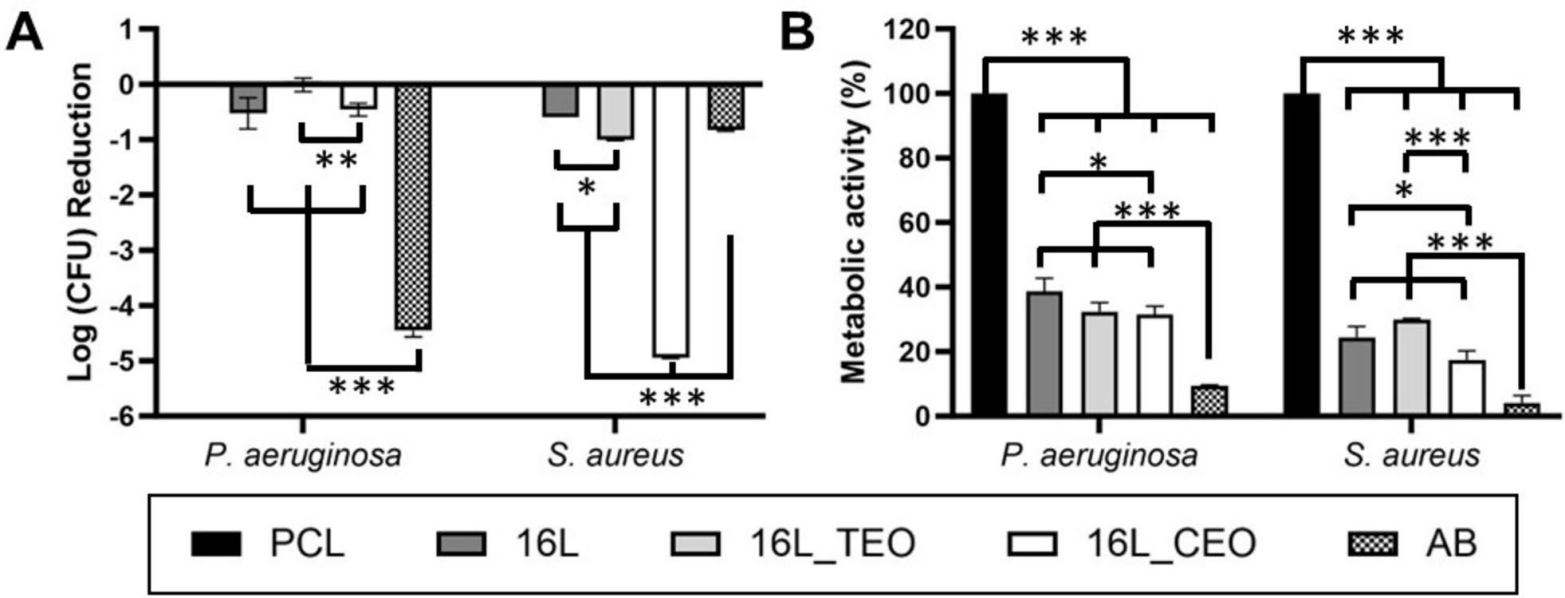
Inactivation (**A**) and metabolic activity (**B**) of *P. aeruginosa* and *S. aureus* treated with the different electrospun membranes before and after LbL-functionalization. The results are shown as average ± SD after normalization to the PCL (n = 3).

Figure 9A shows inactivation results to the different multilayered nanocoated surfaces (images of the counts of colony forming units are reported in Figure S3). TEO exerts its antimicrobial activity through disruption of the bacterial cell wall, reducing cell membrane integrity^66^; antimicrobial activity can vary against different bacterial species, and conflicting results have been reported regarding greater efficacy against Gram-positive versus Gram-negative organisms^67^. Indeed, differences in antimicrobial activity have been noted to individual strains of the same species, as noted in the case of *P. aeruginosa*^66^. In the current study, TEO-functionalized LbL membranes showed no increase in antimicrobial activity relative to the non-functionalized PCL control against *P. aeruginosa* DSMZ 19,880, a strain originally isolated from an infected wound. In the case of *S. aureus*, the TEO-functionalized LbL membrane caused an approximately 1-log reduction of BAA-2313, a clinically derived methicillin-resistant *S. aureus* (MRSA) isolate. In contrast, CEO-functionalized LbL membranes showed increased antimicrobial activity against both bacteria in this study. In the case of *P. aeruginosa*, inactivation was lower, accounting for approximately 50% of the population. However, this was a roughly 5-log reduction in the case of *S. aureus*. Compared with the gentamicin control, the CEO-functionalized LbL membrane showed notably increased inactivation of *S. aureus*. This highlights the potential beneficial applications of EOs, instead of traditional antibiotic-based approaches. Treatment of *S. aureus* infection with antibiotics such as gentamicin are complicated by sub-populations of persister cells within pathogen populations, which show reduced metabolic activity and associated resistance to antimicrobial agents^68^. For antimicrobials that interfere with cellular functions, such as gentamicin, which targets the bacterial ribosome and interferes with protein translation, persister cells showed increased resistance. Since EOs do not only interfere with metabolic function, but rather target the cell wall structure in their mode of action, these may present attractive alternative treatment approaches for use in applications such as functionalized LbL membranes^69^. Analysis of cell metabolic activity also demonstrated the antimicrobial effects of the functionalized LbL membranes (Fig. 9B).

Overall, EO-functionalized LbL membranes showed increased antimicrobial activity against the Gram-positive *S. aureus*, relative to the Gram-negative *P. aeruginosa*, in this study. The results demonstrate functionalization with different EOs show different inhibitory capacity against bacteria. With different antimicrobial components present in EOs (e.g., terpinen-4-ol in TEO, and eugenol in CEO^70^; the spectrum of activity of EO-functionalized LbL membranes could be tailored through the incorporation of different EOs. Further efforts to develop this should consider different strains of bacteria, in addition to different species.

**Fig. 10:**
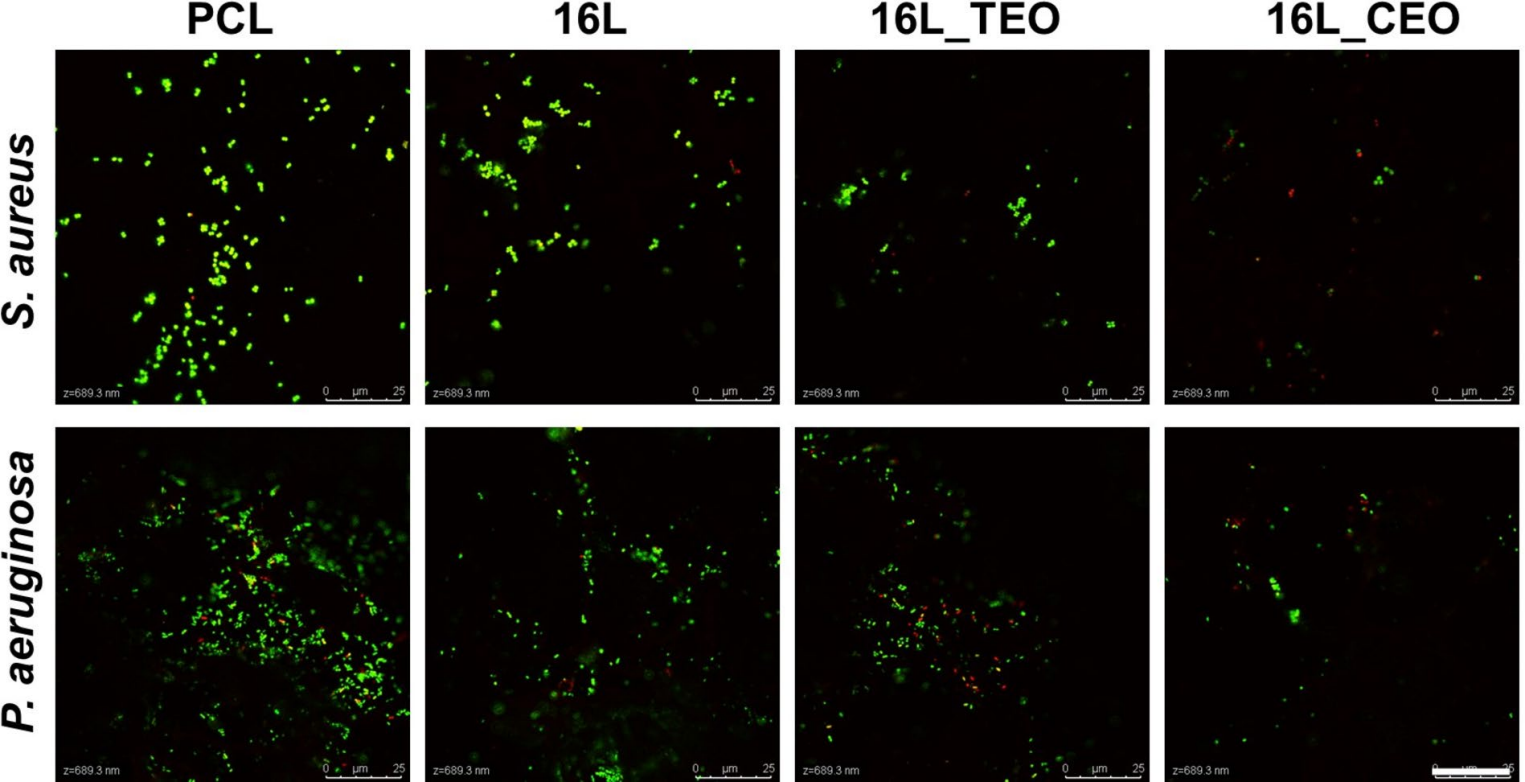
Live/dead staining of (**A**) *S. aureus* and (**B**) *P. aeruginosa* bacteria on the surface treated with the different electrospun membranes before and after LbL-functionalization for 24 h (green―live and red―dead bacteria). Scale bar: 25 μm.

Finally, Live/Dead bacterial staining confirmed the inactivation and metabolic activities results and it was employed to investigate the colonization of both bacterial strains on the surfaces of the membranes through confocal laser scanning microscopy (CLSM) analysis (Fig. 10). The images confirmed the previous bacterial tests with a more robust colonization of *S. aureus* and *P. aeruginosa* bacterial cells on the surfaces of bare PCL samples. The amount of green (live) bacterial cells was notably lower for all the membranes functionalized by LbL assembly, more evident when both EOs were combined with MH, resulting in heightened mortality of both strains.

## Conclusion

In conclusion, this study optimized essential oil nanoemulsions for multilayered nanocoatings and assessed their physicochemical properties and biological interactions. Various extraction methods were systematically evaluated, yielding EO nanoemulsions with sizes suitable for incorporation into nanocoatings, notably cinnamon and tea tree oils ranging from 2 to 10 nm. Physicochemical analyses confirmed monodisperse droplet distributions, and incorporation into nanocoatings maintained membrane integrity. Furthermore, release kinetics over 14 days showed sustained profiles without significant alteration by the nanoemulsions and biological assessments affirmed cytocompatibility with human dermal fibroblasts and upregulation of genes crucial for tissue regeneration. Furthermore, this study has shown that integrating synergically EOs with MH into the multilayered coatings hindered the proliferation of both *S. aureus* and *P. aeruginosa*, making it helpful to the contact-killing of both bacterial strains. These findings underscore the potential of EO nanoemulsions in nanocoatings for biomedical applications, particularly in wound healing and tissue engineering. Further research could include a detailed investigation of the interaction between the polyelectrolytes in the formation of the multilayered coating using atomic force microscopy. Additionally, preclinical validation in, for example, rat models is needed to explore the therapeutic potential of the LbL-functionalized membranes by evaluating wound contraction, tissue regeneration, and long-term healing effects.

## Supplementary Information

Supplementary Information.

## Data Availability

The datasets used and/or analysed during the current study available from the corresponding author on reasonable request.
