# Supplementary material for: Synergistic nanocoating with layer-by-layer functionalized PCL membranes enhanced by manuka honey and essential oils for advanced wound healing
Source: Sci Rep. 2024 Sep 5;14:20715. doi: 10.1038/s41598-024-71466-4 (PMC11377730; doi:10.1038/s41598-024-71466-4)

**Supplementary material**

**Synergistic nanocoating with Layer-by-Layer functionalized PCL membranes enhanced by manuka honey and essential oils for advanced wound healing**

Camilla Gallo, Joel Girón-Hernández, Daisy A Honey, Edward M Fox, Maria Antonia Cassa, Chiara Tonda-Turo, Irene Camagnola, Piergiorgio Gentile

**Table S1.** Values of explanatory variables: essential Oil concentration (% w/v) (X_1_), homogenisation speed (rpm) (X_2_), sonication time (min) (X_3_), and response variable: Size (Y_1_).

| **Run** | **X_1_, oil concentration (% w/v)** | **X_2_, homogenisation speed (rpm)** | **X_3_, *s*onication time (min)** | **Y_1_,**  **TEO size (nm)** | **Y_1_,**  **CEO size (nm)** |
| --- | --- | --- | --- | --- | --- |
| 1 | 4.000 | 10000.0 | 8.000 | 17.9 | 15.5 |
| 2 | 2.000 | 12500.0 | 12.000 | 22.9 | 12.1 |
| 3 | 6.000 | 12500.0 | 4.000 | 18.0 | 27.2 |
| 4 | 4.000 | 10000.0 | 8.000 | 18.4 | 25.4 |
| 5 | 2.000 | 7500.0 | 12.000 | 77.5 | 57.7 |
| 6 | 6.000 | 7500.0 | 12.000 | 55.1 | 44.3 |
| 7 | 6.000 | 7500.0 | 4.000 | 40.8 | 18.1 |
| 8 | 4.000 | 10000.0 | 8.000 | 19.9 | 16.0 |
| 9 | 6.000 | 12500.0 | 12.000 | 21.2 | 12.8 |
| 10 | 2.000 | 12500.0 | 4.000 | 37.4 | 21.7 |
| 11 | 2.000 | 7500.0 | 4.000 | 40.6 | 32.6 |
| 12 | 4.000 | 10000.0 | 8.000 | 21.6 | 13.6 |
| 13 | 4.000 | 14082.5 | 8.000 | 22.8 | 21.5 |
| 14 | 4.000 | 10000.0 | 14.532 | 18.9 | 32.1 |
| 15 | 4.000 | 5917.5 | 8.000 | 87.6 | 57.4 |
| 16 | 4.000 | 10000.0 | 1.468 | 22.1 | 18.7 |
| 17 | 7.266 | 10000.0 | 8.000 | 23.1 | 19.9 |
| 18 | 4.000 | 10000.0 | 8.000 | 19.1 | 16.2 |
| 19 | 0.734 | 10000.0 | 8.000 | 18.5 | 9.8 |
| 20 | 4.000 | 10000.0 | 8.000 | 18.6 | 15.1 |

The optimised conditions for the TEO nanoemulsions were as follows: 7.266% w/v of oil concentration, 12515 rpm of homogenisation speed and a sonication time of 14.5 minutes.

The optimised conditions for the CEO nanoemulsions were as follows: 7.266% w/v of oil concentration, 13635 rpm of homogenisation speed and a sonication time of 14.5 minutes.

**Table S2.** PDI evaluation of the nanoemulsions obtained from the 20 experimental runs indicated by the DOE.

| **Run** | **TEO PDI**  **(%)** | **CEO PDI**  **(%)** |
| --- | --- | --- |
| 1 | 26.4 | 24.2 |
| 2 | 22.2 | 27.6 |
| 3 | 25.7 | 25.4 |
| 4 | 27.5 | 24.5 |
| 5 | 24 | 24.1 |
| 6 | 26.9 | 25.4 |
| 7 | 28.3 | 25.3 |
| 8 | 27.8 | 25.3 |
| 9 | 27.2 | 25.8 |
| 10 | 23.7 | 25.5 |
| 11 | 24.8 | 25.2 |
| 12 | 24.4 | 25.1 |
| 13 | 26.9 | 24.4 |
| 14 | 28.5 | 25.2 |
| 15 | 27.9 | 25.4 |
| 16 | 26.7 | 25 |
| 17 | 28.3 | 25.9 |
| 18 | 26.4 | 24.1 |
| 19 | 8.43 | 27.1 |
| 20 | 27.2 | 24.8 |

**Figure S1.** SEM images of the PCL electrospun membranes at different magnifications X2500 (**A**), X13000 (**B**) and X25000 (**C**).


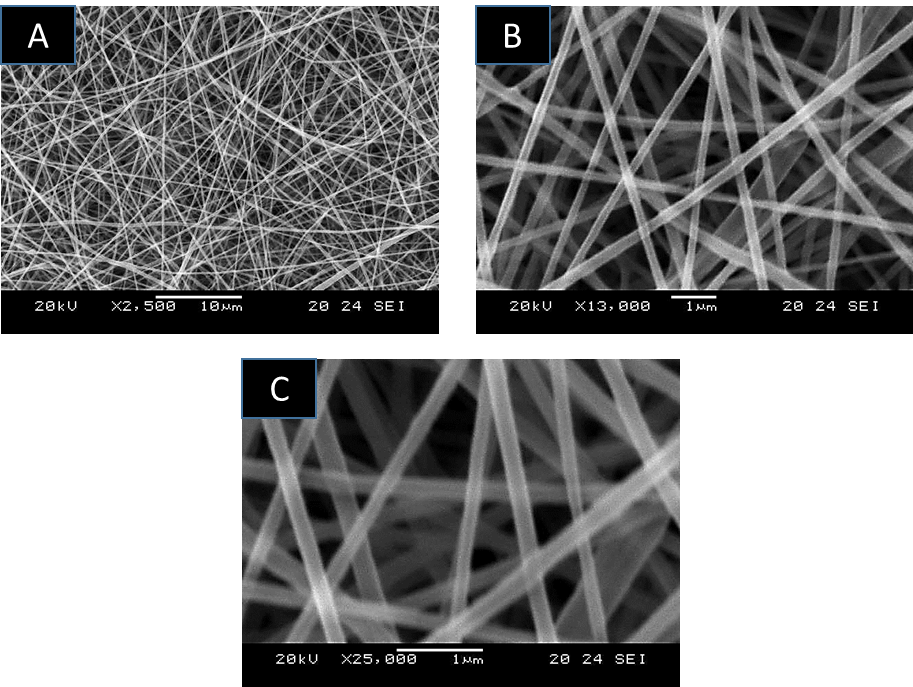


**Figure S2**. Live/Dead image of the human neonatal dermal fibroblast cells seeded on the tissue culture plates as control. Scale bar = 300 μm.


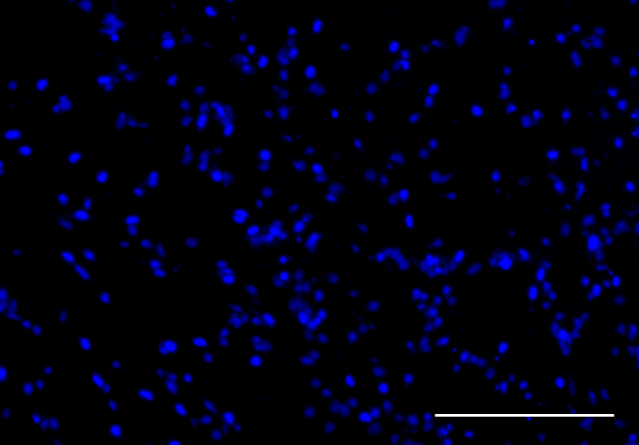


**Figure S3.**

**Figure S4**. Counts of colony forming units resulting after reduction assay of *P. aeruginosa* and *S. aureus* after 24h incubation at 37 °C of LbL-functionalised membranes.


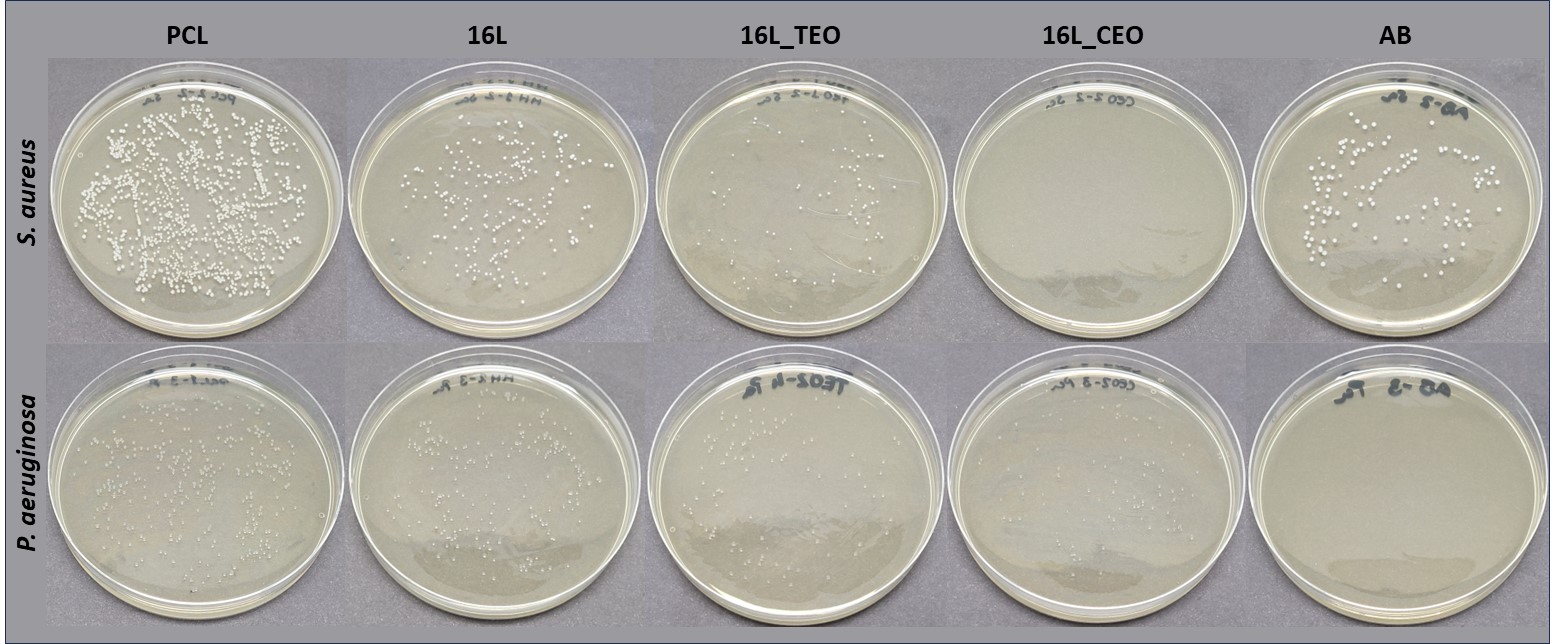

Supplement: Supplementary file 1 — Supplementary Information. [file 41598_2024_71466_MOESM1_ESM.docx]
